# Muscle strength characteristics and the predictive value of handgrip strength in young obese males: A multi‑joint isokinetic and LASSO regression analysis

**DOI:** 10.1371/journal.pone.0353228

**Published:** 2026-07-02

**Authors:** Sen Yang, Jian Li, Shuwei Chen, Xinbi Zhang, Jia Zeng

**Affiliations:** 1 Institute for Sport Performance and Health Promotion, Capital University of Physical Education and Sports, Beijing, China; 2 School of Kinesiology and Health, Capital University of Physical Education and Sports, Beijing, China; 3 Department of Physical Education and Research, Fuzhou University, Fuzhou, China; Children Hospital Lahore: University of Child Health Sciences, PAKISTAN

## Abstract

Obesity is a primary global public health concern, often associated with impaired muscle function and an increased risk of exercise-related injuries. This cross-sectional study aimed to elucidate the associations among muscular strength, body load, and functional capacity in young adults with obesity. A total of 75 male university students were recruited, including a normal-weight group (n = 47, BMI 18.5–24.99) and an obese group (n = 28, BMI ≥ 30). Assessments included handgrip strength, one-repetition maximum (1RM) for squat, deadlift, and bench press, as well as isokinetic strength evaluations across four major joints (shoulder, elbow, hip, and knee), yielding 32 strength-related variables. After normalizing all strength measures to body weight, the obese group demonstrated significantly lower relative strength across all parameters compared to the normal-weight group (*P* < 0.001), indicating a distinct “strength-to-load imbalance.” To identify key predictors of handgrip strength while addressing potential overfitting, we employed least absolute shrinkage and selection operator (LASSO) regression with cross-validation. The final model retained five predictors: elbow flexion maximum work at 180°/s (EF180°Wmax), elbow flexion peak torque at 180°/s (EF180°Fmax), elbow extension peak torque at 60°/s (EE60°Fmax), shoulder flexion peak torque at 60°/s (SF60°Fmax), and group (obese vs. normal), together explaining 63.3% of the variance in handgrip strength (adjusted R² = 0.606), although this finding should be interpreted with caution due to the sample size and requires validation in larger cohorts. Notably, group membership remained a significant independent predictor (β = −0.23, P = 0.014), underscoring the persistent deficit in relative strength associated with obesity even after accounting for joint-specific strength. By incorporating a multidimensional assessment of strength across multiple joints, this study moves beyond the limitations of single-joint evaluations and offers theoretical support for the potential use of handgrip strength as a practical indicator of overall muscle function in obese populations. Further research with larger samples and longitudinal designs is needed to validate its utility as a screening tool. These findings have important implications for designing function-oriented, precision-based exercise interventions for obese populations.

## Introduction

Obesity is a prominent global public health issue. The World Health Organization defines it as a chronic and complex disease characterized by excessive fat accumulation, which increases the risk of metabolic disorders, cardiovascular disease, and at least 13 types of cancer. Abnormal body composition in individuals with obesity is also associated with impaired motor function and a heightened risk of exercise-related injuries [1,2]. The rising prevalence of obesity is linked to increased risks of hyperuricemia, diabetes, chronic kidney disease, and cardiovascular conditions [3–7]. Moreover, obesity has been shown to negatively affect skeletal muscle across all life stages, including adolescence, young adulthood, and older age [8–11].

Muscle strength is a critical indicator of physical fitness and is closely associated with functional ability, athletic performance, and the risk of chronic disease. Numerous studies have shown that individuals with obesity exhibit higher absolute muscular strength than those of normal weight. However, after normalization by body weight, their relative strength is notably lower [12,13]. This “strength-to-load imbalance” has been linked to an increased risk of all-cause mortality. Cohort studies have reported that each 5 kg increase in handgrip strength is associated with a 31% reduction in all-cause mortality risk (95% CI, 0.64–0.74), and a 10% increase in quadriceps strength is associated with a 14% reduction in mortality (95% CI, 0.80–0.93) [14,15]. Higher handgrip strength has also been inversely associated with cardiovascular and cancer mortality [16], while upper- and lower-limb strength, as measured by bench press and leg press, respectively, show similar associations with reduced cancer mortality risk [17].

Isokinetic strength testing is a reliable method for assessing muscle force production and neuromuscular control, and it enhances joint stability, flexibility, and motor coordination [18,19]. It is regarded as one of the most valuable tools for evaluating muscular strength and joint range of motion [18], with particularly high reliability in lower limb assessments [20,21]. Recent research has further demonstrated the effectiveness of isokinetic assessments in identifying and correcting muscle imbalances, particularly in the shoulder musculature [22]. Moreover, studies investigating obese populations have shown that these individuals demonstrate disadvantages in maximal strength, muscular endurance, and joint stability. For instance, in obese older men, handgrip strength has been found to show weak-to-moderate correlations with knee extensors and flexors (*p* < 0.05) [23]. Another study comparing quadriceps function between obese and normal-weight males found that the obese group had significantly higher torque loss (*P* < 0.05), along with 20% greater absolute strength and power (*P* < 0.01); however, when normalized to body weight, their values were 32% lower (*P* < 0.001) [10]. Additionally, absolute torque of extension and flexion in individuals with severe obesity was weakly to moderately correlated with total lean mass and moderately correlated with lower limb lean mass, suggesting that improving absolute torque and regional lean mass—particularly in the lower limbs—may help prevent functional decline and loss of physical capacity [24].

Notably, most existing studies have focused on single joints (e.g., knee or ankle), often overlooking the dynamic interrelationships between different strength qualities and multi-joint loading characteristics [12,25]. Furthermore, the specific effects of obesity on muscle function across different joints and whether handgrip strength can serve as a valid proxy for overall muscular fitness remain insufficiently understood. Emerging evidence suggests that handgrip strength reflects not only upper-limb function but may also be closely associated with global muscular strength. Therefore, investigating the relationships between handgrip strength and performance in exercises such as squat, deadlift, and bench press, as well as isokinetic strength across multiple joints, could offer critical insights for refining muscle strength assessment methods and developing targeted exercise interventions for individuals with obesity. This has important implications for injury prevention and improving functional performance in clinical and athletic settings.

## Materials and methods

### Participants

This cross-sectional study recruited 75 male university students from Capital University of Physical Education and Sports in Beijing. Participants were categorized based on the World Health Organization’s BMI classification: underweight (BMI < 18.49), normal weight (18.5 ≤ BMI < 24.99), overweight (25 ≤ BMI < 29.99), and obese (BMI ≥ 30). Two groups were included for comparison in this study: a normal-weight group (n = 47, BMI 18.5–24.99) and an obese group (n = 28, BMI ≥ 30) (Table 1). The inclusion criteria required participants to be male and aged 20–30 years. The normal-weight group had a mean age of 22.34 ± 2.82 years, and the obese group had a mean age of 22.67 ± 1.74 years (Table 1). Exclusion criteria included the presence of contraindications to physical activity, cardiovascular disease, diabetes, chronic illnesses, infectious diseases such as hepatitis B or tuberculosis, long-term smoking habits, and regular participation in structured resistance training (≥2 sessions per week) within the past six months. Participants with any musculoskeletal injuries affecting the upper or lower extremities in the preceding six months were also excluded. The recruitment period for participants was from June 1, 2024, to June 30, 2024. The Ethics Committee of Capital University of Physical Education and Sports approved the study protocol and testing procedures. Prior to participation, all individuals received both verbal and written explanations detailing the study’s objectives, testing procedures, potential risks, and their right to withdraw at any time without penalty. Participants were given sufficient time to consider the information and ask questions. Written informed consent was obtained from all participants before any data collection commenced, in accordance with the ethical guidelines of the Capital University of Physical Education and Sports and the Declaration of Helsinki. The study was approved by the Ethics Committee of the Capital University of Physical Education and Sports and registered with the Chinese Clinical Trial Registry (ChiCTR2400089154).

**Table 1:** Basic characteristics of the participants.

Group	Age (years)	Height (m)	Body Mass (kg)	Body Weight (N)	BMI (kg/m²)
Normal (n = 47)	22.34 ± 2.82	1.78 ± 0.06	73.38 ± 7.14	719.08 ± 69.95	23.11 ± 1.43
	(21.54–23.15)	(1.76–1.80)	(71.33–75.42)	(699.08–739.08)	(22.70–23.52)
Obese (n = 28)	22.67 ± 1.74	1.77 ± 0.06	101.84 ± 7.24	998.02 ± 70.97	32.35 ± 1.56
	(22.03–23.32)	(1.75–1.80)	(99.16–104.52)	(971.73–1024.31)	(31.77–32.92)
*t value*	−0.55	0.42	−16.61	−16.61	−26.12
*p value*	0.582	0.677	<0.001**	<0.001**	<0.001**
Cohen’s *d*	−0.13	0.10	−3.97	−3.97	−6.24

**Note**: Data are presented as mean±standard deviation (95% confidence interval). Cohen’s *d* effect size: negative values denote higher values in the obese group. *p*-values are from independent t‑tests. * *p* < 0.05 ** *p* < 0.01.

### Experimental procedures

#### Handgrip strength.

Handgrip strength was assessed using a standard electronic hand dynamometer, which was calibrated and confirmed to be functioning properly before testing. Participants stood upright with arms relaxed by their sides and elbows flexed at approximately 90°, with the palm facing the body. The dynamometer handle was aligned with the fingers, ensuring full contact between the hand and the grip surface. Each trial began with the right hand, followed by the left. Participants were instructed to squeeze the dynamometer with maximal effort, aiming to reach peak grip strength as quickly as possible. Three trials were performed for each hand, and the highest value was recorded for analysis. A 30-second rest interval was provided between trials to minimize fatigue-related interference.

#### One-Repetition Maximum (1RM).

Participants were instructed to avoid vigorous physical activity within 24 hours before testing. Before attempting 1RM, they were taught the correct techniques for the bench press, squat, and deadlift exercises. To ensure proper technique and minimize injury risk, all participants attended a familiarization session 3–7 days before the 1RM testing day. During this session, they received detailed instructions and supervised practice on the correct techniques for the bench press, squat, and deadlift exercises, following standardized protocols [26]. On the testing day, a standardized warm-up was performed consisting of 5 minutes of light cycling, dynamic stretching, and two submaximal sets of each exercise: first set with 5–10 repetitions at 40–60% of estimated 1RM, and second set with 3–5 repetitions at 60–80% of estimated 1RM. The 1RM test began with a predicted load and progressively increased by 5–10% for successful attempts until the participant could no longer complete a full repetition with proper form. All attempts were supervised by certified strength and conditioning specialists, with safety spotters present and a power rack used for the squat to ensure participant safety. The highest successfully lifted weight with proper technique was recorded as the 1RM. Typically, 1RM was determined within 3–5 attempts, with a rest period of 3–5 minutes between each attempt to ensure full recovery. The relationship between body mass and 1RM performance has been established in male athletes, supporting the validity of these assessments across different body compositions [27].

#### Isokinetic strength testing.

Isokinetic muscle strength was evaluated using the Isomed 2000 Basic System (DR Ferstl GmbH, Germany), focusing on flexion and extension at the knee and hip joints (lower limbs) and the elbow and shoulder joints (upper limbs). Methodological considerations in strength assessment protocols are crucial for ensuring valid and reliable measurements, particularly when using force-based evaluations [28]. Functional strength assessments have demonstrated high intrasession reliability and external responsiveness, supporting their use in comprehensive strength evaluation protocols [29]. Before testing, the Isomed 2000 system was calibrated according to the manufacturer’s specifications. Gravity compensation was performed individually for each participant at the start of the testing session. With the participant’s limb completely relaxed, the passive torque exerted by the limb’s weight was measured by the dynamometer. This value was automatically recorded by the system software and subtracted from all subsequent torque measurements during maximal voluntary contractions, ensuring that the reported values reflect only active muscle force production. This compensation procedure was applied once per participant and remained active for all subsequent joint and velocity conditions. After 3–5 familiarization trials to ensure proper execution and reduce learning effects, the formal testing commenced. Two angular velocities were assessed: slow (60°/s) and fast (180°/s), using a concentric/concentric contraction mode. Each participant completed 3 sets per joint. At 60°/s, each set included 5 repetitions; at 180°/s, each set included 20 repetitions. A 5-minute rest interval was provided between different velocity conditions [30]. The average peak torque across repetitions was used for statistical analysis. The joints tested and parameter configurations are summarized in Table 2.

**Table 2:** Joint and Parameter Settings for Isokinetic Strength Testing.

Joint (Dominant Side)	Contraction Mode	Angular Velocity	Repetitions	Sets	Rest Between Sets
Knee Joint	Concentric/Flexion–Extension	60º/s	5	3	2 min
	Concentric/Flexion–Extension	180º/s	20	3	2 min
Hip Joint	Concentric/Flexion–Extension	60º/s	5	3	2 min
	Concentric/Flexion–Extension	180º/s	20	3	2 min
Elbow Joint	Concentric/Flexion–Extension	60º/s	5	3	2 min
	Concentric/Flexion–Extension	180º/s	20	3	2 min
Shoulder Joint	Concentric/Flexion–Extension	60º/s	5	3	2 min
	Concentric/Flexion–Extension	180º/s	20	3	2 min

**Note:** Testing procedures strictly followed the Isomed 2000 system’s standard operating protocol.

### Statistical analysis

All statistical analyses were performed using MATLAB R2025b (MathWorks, Natick, MA, USA). Continuous variables were expressed as mean ± standard deviation ($\bar{x}$ ± s). Normality was assessed using the Shapiro–Wilk test, and homogeneity of variances was examined using Levene’s test. For each variable, group comparisons between normal-weight (n = 47) and obese (n = 28) participants were conducted using independent t‑tests (if normality and homogeneity assumptions were met) or Mann–Whitney U tests (if assumptions were violated). Effect sizes were calculated as Cohen’s *d* for t‑tests and as rank‑biserial correlation *r* for Mann–Whitney tests, along with their 95% confidence intervals. To account for multiple comparisons across the 37 strength‑related variables (32 isokinetic, 2 handgrip, 3 one‑repetition maximum), the Benjamini–Hochberg false discovery rate (FDR) correction was applied, with statistical significance set at FDR‑adjusted *p* < 0.05. Predictive modeling for handgrip strength was performed using LASSO regression with 10‑fold cross‑validation. The LASSO model included all 32 isokinetic variables and group (normal‑weight vs. obese) as predictors, with HGS-R as the dependent variable. The optimal regularization parameter (lambda) was selected using the one standard error rule (lambda1SE). Variables with non‑zero coefficients at lambda1SE were retained and subsequently entered into a multiple linear regression model to obtain conventional statistics (unstandardized and standardized coefficients, 95% confidence intervals, and variance inflation factors). All tests were two‑tailed.

## Results

### Participant characteristics

The basic characteristics of the participants are presented in Table 1. There were no significant differences between the normal-weight and obese groups in age (22.34 ± 2.81 vs. 22.67 ± 1.74 years, p = 0.582) or height (1.78 ± 0.06 vs. 1.77 ± 0.06 m, p = 0.677). As expected, the obese group had significantly higher body mass, body weight, and BMI (all p < 0.001).

### Comparison of normalized strength measures between groups

Group comparisons for all 37 body‑weight normalized strength variables are shown in Table 3. The normal‑weight group exhibited significantly higher values than the obese group across every measure, including isokinetic peak torque and work at all four joints (shoulder, elbow, hip, knee) at both angular velocities (60°/s and 180°/s), handgrip strength (right and left), and one‑repetition maximum (1RM) for squat, deadlift, and bench press. After applying Benjamini–Hochberg false discovery rate (FDR) correction, all differences remained statistically significant (FDR‑adjusted *p* < 0.05; Table 3). Effect sizes ranged from moderate to large (Cohen’s *d* = 0.41–1.68 for independent t‑tests; rank‑biserial *r* = 0.41–0.67 for Mann–Whitney tests), indicating substantial deficits in muscle strength associated with obesity. Detailed results, including means, standard deviations, test statistics, effect sizes with 95% confidence intervals, and FDR‑adjusted *p*‑values, are presented in Table 3. Participants in the normal-weight group exhibited superior performance in speed endurance and peak torque at the elbow, shoulder, knee, and hip joints, as well as in handgrip strength and 1RM tests, indicating that obesity is associated with both reduced muscular strength and endurance. Previous studies have reported that individuals with obesity tend to have lower handgrip strength, lower limb strength, and trunk strength compared to their normal-weight counterparts. This may be partially attributed to the secretion of pro-inflammatory factors by adipose tissue—such as leptin resistance and insulin resistance—which can interfere with muscle metabolism, leading to decreased protein synthesis and increased protein degradation. These physiological disruptions can result in diminished muscle mass and strength performance [31]. Taken together, these findings suggest that individuals with obesity have disadvantages in muscle strength, endurance, and overall physical performance, highlighting the need for targeted interventions.

**Table 3:** Comparison of body‑weight normalized strength measures between normal‑weight and obese groups.

Variable	Normal (n = 47)	Obese (n = 28)	Effect Size (95% CI)	Test	*p*_FDR_
**Elbow**					
EE180°Wmax	0.61 ± 0.17	0.45 ± 0.13	*d*=1.04 (0.53–1.54)	t‑test	5.58281E-05
EF180°Wmax	0.58 ± 0.14	0.42 ± 0.11	*d*=1.26 (0.74–1.78)	t‑test	2.92916E-06
EE180°Fmax	0.51 ± 0.13	0.38 ± 0.09	*r*=0.46 (0.27–0.63)	MW	7.11065E-05
EF180°Fmax	0.48 ± 0.13	0.35 ± 0.08	*r*=0.47 (0.27–0.63)	MW	5.74083E-05
EE60°Wmax	0.78 ± 0.18	0.56 ± 0.21	*r*=0.53 (0.34–0.67)	MW	9.36638E-06
EF60°Wmax	0.77 ± 0.18	0.55 ± 0.16	*d*=1.28 (0.77–1.80)	t‑test	2.31126E-06
EE60°Fmax	0.58 ± 0.15	0.42 ± 0.12	*d*=1.13 (0.62–1.63)	t‑test	1.90295E-05
EF60°Fmax	0.54 ± 0.14	0.37 ± 0.09	*r*=0.57 (0.39–0.71)	MW	2.47657E-06
**Shoulder**					
SE180°Wmax	0.98 ± 0.29	0.61 ± 0.21	*r*=0.57 (0.39–0.71)	MW	2.41788E-06
SF180°Wmax	0.78 ± 0.21	0.63 ± 0.14	*d*=0.78 (0.29–1.27)	t‑test	0.001674198
SE180°Fmax	0.86 ± 0.22	0.51 ± 0.16	*r*=0.67 (0.52–0.78)	MW	8.18284E-08
SF180°Fmax	0.76 ± 0.18	0.54 ± 0.12	*r*=0.59 (0.42–0.72)	MW	1.41887E-06
SE60°Wmax	1.25 ± 0.33	0.81 ± 0.21	*r*=0.63 (0.48–0.75)	MW	2.08614E-07
SF60°Wmax	1.07 ± 0.24	0.87 ± 0.16	*r*=0.41 (0.20–0.58)	MW	0.000288032
SE60°Fmax	0.82 ± 0.19	0.54 ± 0.13	*r*=0.67 (0.52–0.78)	MW	6.99027E-08
SF60°Fmax	0.73 ± 0.14	0.56 ± 0.11	*d*=1.37 (0.84–1.89)	t‑test	9.8464E-07
**Knee**					
KE180°Wmax	1.85 ± 0.34	1.52 ± 0.27	*r*=0.48 (0.29–0.64)	MW	3.69037E-05
KF180°Wmax	1.04 ± 0.21	0.79 ± 0.21	*d*=1.17 (0.66–1.68)	t‑test	9.78677E-06
KE180°Fmax	1.97 ± 0.34	1.72 ± 0.31	*d*=0.73 (0.25–1.22)	t‑test	0.003023543
KF180°Fmax	1.25 ± 0.28	1.03 ± 0.27	*d*=0.81 (0.32–1.30)	t‑test	0.001254743
KE60°Wmax	2.49 ± 0.45	1.81 ± 0.34	*d*=1.64 (1.09–2.18)	t‑test	3.53015E-08
KF60°Wmax	1.38 ± 0.28	0.99 ± 0.24	*d*=1.50 (0.97–2.04)	t‑test	1.25304E-07
KE60°Fmax	2.71 ± 0.54	2.10 ± 0.40	*d*=1.23 (0.72–1.74)	t‑test	4.57209E-06
KF60°Fmax	1.48 ± 0.30	1.15 ± 0.29	*d*=1.10 (0.59–1.61)	t‑test	2.4303E-05
**Hip**					
HE180°Wmax	2.66 ± 0.97	1.66 ± 0.59	*r*=0.50 (0.31–0.66)	MW	2.04502E-05
HF180°Wmax	1.34 ± 0.35	0.98 ± 0.29	*r*=0.49 (0.30–0.65)	MW	2.6905E-05
HE180°Fmax	3.39 ± 0.93	2.44 ± 0.70	*d*=1.10 (0.60–1.61)	t‑test	2.46461E-05
HF180°Fmax	1.84 ± 0.52	1.54 ± 0.49	*d*=0.59 (0.11–1.07)	t‑test	0.015746397
HE60°Wmax	3.68 ± 0.98	2.30 ± 0.70	*d*=1.55 (1.02–2.09)	t‑test	6.35693E-08
HF60°Wmax	1.92 ± 0.47	1.33 ± 0.31	*r*=0.58 (0.41–0.71)	MW	1.71812E-06
HE60°Fmax	4.02 ± 1.00	2.80 ± 0.71	*r*=0.55 (0.36–0.69)	MW	4.69611E-06
HF60°Fmax	2.05 ± 0.51	1.63 ± 0.47	*d*=0.84 (0.35–1.33)	t‑test	0.000843222
**Grip strength**					
HGS-R	0.59 ± 0.11	0.45 ± 0.08	*r*=0.59 (0.42–0.72)	MW	1.18014E-06
HGS-L	0.57 ± 0.10	0.41 ± 0.08	*d*=1.68 (1.14–2.23)	t‑test	3.03867E-08
**1RM**					
DL	1.45 ± 0.37	1.03 ± 0.23	*r*=0.54 (0.36–0.68)	MW	5.81619E-06
BS	1.38 ± 0.38	0.98 ± 0.26	*r*=0.49 (0.30–0.65)	MW	2.72132E-05
BP	0.87 ± 0.22	0.61 ± 0.17	*d*=1.27 (0.75–1.79)	t‑test	2.72281E-06

**Note:** Wmax: maximum work, Fmax: peak torque, 180°/60°: angular velocities (°/s), DL: Deadlift, BS: Back Squat, BP: Bench Press, HGS-R: Right Hand Grip Strength, HGS-L: Left Hand Grip Strength, EE: Elbow extension, EF: Elbow flexion, SE: Shoulder extension, SF: Shoulder flexion, KE: Knee extension KF: Knee flexion, HE: Hip extension, HF: Hip flexion. Values are mean ± SD. Effect size: Cohen’s *d for* independent t‑tests; rank‑biserial correlation *r for Mann–Whitney* (MW) tests. 95% confidence intervals are given in parentheses. All *p*_FDR_ values < 0.05 after Benjamini–Hochberg correction. Handgrip and 1RM units: kg/kg; isokinetic torque units: N·m/kg.

### Principal component analysis and interaction testing

To reduce the dimensionality of the 32 isokinetic variables, principal component analysis (PCA) was performed on the full sample (N = 75). The first four principal components explained 80.95% of the total variance and were retained for subsequent analyses. Using these four components, we tested for interactions between group (normal‑weight vs. obese) and joint strength dimensions in predicting HGS-R. No significant interactions were found (all *p > 0.05*), indicating that the relationship between joint strength and grip strength did not differ between groups. Therefore, subsequent regression analyses were conducted on the full sample with group included as a covariate.

### LASSO regression for handgrip strength

To identify the most important predictors of handgrip strength while avoiding overfitting, we applied LASSO regression with 10‑fold cross‑validation. The model included all 32 isokinetic variables and group (normal‑weight vs. obese) as predictors, with HGS-R as the dependent variable. The optimal regularization parameter was selected using the one‑standard‑error rule (lambda1SE).

The LASSO model retained five predictors with non‑zero coefficients: elbow flexion peak torque at 180°/s (EF180°Fmax), elbow flexion maximum work at 180°/s (EF180°Wmax), elbow extension peak torque at 60°/s (EE60°Fmax), shoulder flexion peak torque at 60°/s (SF60°Fmax), and group (obese vs. normal). These variables were subsequently entered into a multiple linear regression model to obtain conventional statistics (Table 4). The final model explained 63.3% of the variance in handgrip strength (adjusted R² = 0.606, F₅,₆₉ = 23.79, p < 0.001). Group membership remained a significant independent predictor (β = −0.23, p = 0.014). Variance inflation factors (VIFs) ranged from 1.57 to 6.26. Although the VIFs for EF180°Wmax (6.26) and EF180°Fmax (5.76) exceeded 5, both variables were retained. In a LASSO‑derived predictive model, moderate multicollinearity does not bias coefficient estimates or impair prediction; it primarily inflates standard errors. Moreover, the two variables represent physiologically distinct constructs (peak torque vs. maximum work) and were both consistently selected by cross‑validation, indicating that each contributes unique information to handgrip strength beyond their shared variance.

**Table 4:** LASSO-derived multiple linear regression model for handgrip strength (right hand) in the full sample (N = 75).

Variable	B (95% CI)	Beta	SE	*t*	*p*	VIF
Constant	0.564*	–	–	–	–	–
EF180°Fmax	0.311 (−0.007, 0.629)	0.336	0.162	1.92	0.059	5.76
EF180°Wmax	0.135 (−0.158, 0.427)	0.165	0.149	0.90	0.370	6.26
EE60°Fmax	0.090 (−0.067, 0.247)	0.118	0.080	1.12	0.266	2.08
SF60°Fmax	0.075 (−0.093, 0.243)	0.096	0.086	0.88	0.383	2.26
Group (obese vs. normal)	−0.057 (−0.101, −0.012)	−0.230	0.023	−2.51	0.014	1.57

Note: B = unstandardized coefficient; Beta = standardized coefficient; SE = standard error; CI = confidence interval; VIF = variance inflation factor. Dependent variable: right handgrip strength (normalized to body weight, kg/kg). Model R^2^ = 0.633, adjusted R^2^ = 0.606, F_5,69_ = 23.79, p < 0.001. * *p* < 0.05.

## Discussion

This cross-sectional study identified associations between obesity and muscle function in young adult males, characterized by a pronounced “strength-to-load imbalance.” The findings suggest that obesity is associated with a “strength-to-load imbalance,” which may be related to a combination of factors, including metabolic dysregulation, abnormal mechanical loading, and neuromuscular adaptations. When normalized to body weight, the obese group demonstrated significantly lower relative strength across all measures, with moderate to large effect sizes (Cohen’s *d* = 0.41–1.68; rank‑biserial *r* = 0.41–0.67). This is consistent with previous studies showing that individuals with obesity exhibit higher absolute but lower relative strength [12,13]. However, due to the cross-sectional design, these potential mechanisms should be interpreted as hypotheses requiring confirmation in future longitudinal or interventional studies.

The lower relative strength in the obese group was particularly marked for the knee extensors and hip flexors (Cohen’s *d* up to 1.64 and 1.55, respectively), which may reflect the chronic mechanical overload imposed on these weight‑bearing muscles. Excessive body mass increases the lever arm torque that these muscles must counteract, potentially leading to altered muscle spindle sensitivity and reduced α‑motoneuron activation [32]. The hip flexor deficit might also be related to gait adaptations commonly observed in obesity, such as shortened stride length and prolonged swing phase [33]. Interestingly, upper limb muscles, especially shoulder flexors, showed relatively preserved performance under high-velocity conditions, suggesting a possible proximal compensation strategy.

Adipose tissue‑derived inflammatory cytokines such as interleukin-6 (IL-6) and tumor necrosis factor-alpha (TNF-α) have been shown to inhibit muscle protein synthesis by suppressing the mTOR signaling pathway [34], and mitochondrial dysfunction in skeletal muscle among obese individuals compromises energy metabolism efficiency [35]. These systemic factors may contribute to the lower normalized strength observed here. Furthermore, the poorer performance at 180°/s in the obese group is consistent with evidence of a reduced proportion of type II muscle fibers [36] and impaired neural drive during rapid contractions [37]. Although these mechanisms were not directly tested in this study, they offer plausible explanations.

To move beyond single‑joint comparisons, we used principal component analysis to reduce the 32 isokinetic variables to four composite dimensions (explaining 80.95% of the variance). No significant interactions between these dimensions and group were found in predicting handgrip strength, indicating that the relationship between joint strength and grip strength is similar in normal‑weight and obese individuals. We therefore employed LASSO regression on the full sample (N = 75) to identify robust predictors of handgrip strength while controlling for overfitting. The final model retained five predictors: elbow flexion peak torque at 180°/s (EF180°Fmax), elbow flexion maximum work at 180°/s (EF180°Wmax), elbow extension peak torque at 60°/s (EE60°Fmax), shoulder flexion peak torque at 60°/s (SF60°Fmax), and group (obese vs. normal), together explaining 63.3% of the variance in handgrip strength (adjusted R² = 0.606). Group remained a significant independent predictor (β = −0.23, p = 0.014), underscoring the persistent deficit in relative strength associated with obesity even after accounting for joint‑specific strength.

The selected elbow and shoulder variables highlight the importance of the upper limb kinetic chain in grip production. EE180°Fmax contributed the largest standardized coefficient (β = 0.336), consistent with the role of elbow flexors as prime movers in gripping tasks. The inclusion of both slow (60°/s) and fast (180°/s) angular velocities suggests that grip strength depends on a combination of maximal force production and explosive capacity. These findings provide an empirical basis for targeted training strategies: interventions aiming to improve handgrip strength in obese individuals should emphasize dynamic elbow flexion exercises (e.g., fast biceps curls) and shoulder flexion stability work, while also addressing the global deficit in relative strength through whole‑body resistance training.

### Limitations and future perspectives

This study has several limitations. First, the sample consisted solely of young adult male university students, lacking representation from females and other age groups, which limits the generalizability of the findings. Second, due to its cross-sectional design, this study cannot establish causal relationships between obesity and muscle strength characteristics; longitudinal research is needed to determine temporal dynamics. Third, although we applied Benjamini–Hochberg FDR correction to account for multiple comparisons and used LASSO regression with cross‑validation to mitigate overfitting, the sample size (particularly n = 28 in the obese group) remains modest for some multivariate analyses. The results of the regression model should be interpreted with caution and require validation in larger, independent cohorts. Fourth, the absence of micro-level indicators, such as muscle mass and muscle fiber type composition, restricted the exploration of the structural basis underlying strength differences. Moreover, key neural control mechanisms—such as motor unit recruitment strategies and corticospinal excitability—were not included in the analytical framework, preventing a comprehensive understanding of how obesity influences neuromuscular regulation of strength. Fifth, the PCA conducted for interaction testing was performed on 32 isokinetic variables with a sample size of 75 participants, yielding a participant-to-variable ratio of approximately 2.3:1. This ratio falls below the generally accepted minimum threshold for stable dimensionality reduction. Therefore, the stability of the extracted components should be interpreted with caution, and the results of the PCA-based interaction tests require validation in larger, independent cohorts. To address these limitations, future research should adopt multi-center, multi-population cohort designs and integrate technologies such as radiomics and surface electromyography (sEMG) to build a multidimensional assessment system, ultimately enhancing the mechanistic understanding of obesity-related muscular impairments.

Future research should focus on developing multidimensional assessment and intervention frameworks. A multi-modal evaluation system that integrates technologies such as dual-energy X-ray absorptiometry (DEXA), electromyography (EMG), and functional magnetic resonance imaging could be employed to explore the biomechanical, metabolic, and neuroregulatory mechanisms by which obesity affects muscular strength across different scales. In addition, the development of wearable smart monitoring devices may enable real-time tracking of the dynamic balance between force generation and mechanical loading. Investigating the synergistic effects of metabolic interventions—such as gut microbiota modulation—and resistance training could provide insights for tailoring individualized exercise programs for metabolically healthy versus unhealthy obese phenotypes. Furthermore, muscular function assessment should be incorporated into clinical obesity management pathways, with relative handgrip strength, squat power, and similar functional indices serving as core components of precision prevention systems. This shift from a weight-centric to a function-oriented paradigm would offer a new approach to preventing and managing obesity-related functional impairments.

## Conclusion

Young adults with obesity demonstrate significantly lower body‑weight‑normalized strength across multiple joints compared to their normal‑weight peers. Handgrip strength is associated with upper limb joint torques, particularly elbow and shoulder flexors, and the persistence of a negative group effect highlights the global nature of the strength deficit. These findings support the potential utility of handgrip strength as a practical indicator of overall muscle function in obesity‑related research. Further studies with larger, more diverse samples and prospective designs are needed to validate this marker and to develop effective exercise interventions.

## Supporting information

S1 Table Basic information descriptive statistics. (XLSX)

S2 Table GroupComparison_Results_Updated. (XLSX)

S3 Table LASSO_Regression_Results. (XLSX)
